# Statistical distributions of optimal global alignment scores of random protein sequences

**DOI:** 10.1186/1471-2105-6-257

**Published:** 2005-10-15

**Authors:** Hongxia Pang, Jiaowei Tang, Su-Shing Chen, Shiheng Tao

**Affiliations:** 1 School of Life Science, Northwest A&F University, Yangling, Shaanxi, China; 2 Institute of Bioinformatics, Northwest A&F University, Yangling, Shaanxi, China

## Abstract

**Background:**

The inference of homology from statistically significant sequence similarity is a central issue in sequence alignments. So far the statistical distribution function underlying the optimal global alignments has not been completely determined.

**Results:**

In this study, random and real but unrelated sequences prepared in six different ways were selected as reference datasets to obtain their respective statistical distributions of global alignment scores. All alignments were carried out with the Needleman-Wunsch algorithm and optimal scores were fitted to the Gumbel, normal and gamma distributions respectively. The three-parameter gamma distribution performs the best as the theoretical distribution function of global alignment scores, as it agrees perfectly well with the distribution of alignment scores. The normal distribution also agrees well with the score distribution frequencies when the shape parameter of the gamma distribution is sufficiently large, for this is the scenario when the normal distribution can be viewed as an approximation of the gamma distribution.

**Conclusion:**

We have shown that the optimal global alignment scores of random protein sequences fit the three-parameter gamma distribution function. This would be useful for the inference of homology between sequences whose relationship is unknown, through the evaluation of gamma distribution significance between sequences.

## Background

Sequence alignment is a central problem in computational molecular biology. Every branch of molecular biology- from database search, phylogeny reconstruction to protein structure prediction- takes sequence alignments as its foundation. The functional and/or structural properties of a new sequence could be predicted from its degree of similarity with some clearly defined and known sequences. If the similarity between two sequences is statistically significant and does not simply stem from sequence repeats of low complexity, then the two sequences are likely to be homologous and thus may have similar functions and/or structures.

To understand whether an observed sequence similarity implies indeed a functional or evolutionary link, or is just a chance event is the central problem for the evaluation of the statistical significance of sequence alignment scores. The basic question to be answered is: what is the probability that a similarity score as great as that actually observed in a comparison between real sequences could have arisen by chance, when sampling from suitably-defined populations of random unrelated sequences? This question is generally addressed by analyzing the distribution of optimal alignment scores from random or real but unrelated sequences [1], which is the approach applied in this research.

Accurate statistical estimate for similarity searches for local alignments has been studied comprehensively [1-4], and the Gumbel distribution is used to estimate the statistical significance for local alignments [5]. However, we still lack a complete theoretical solution to the optimal global alignment between sequences, due to the fact that global alignment scores grow proportionally to the length of the sequences and small changes in the scoring system can produce a different alignment [6].

Abagyan and Batalov suggested that global alignment scores between unrelated protein sequences followed the Gumbel distribution [7]. However, since the scoring system that they used favoured local alignments, these alignments they produced may not be global but local. Dayhoff *et al *(1978) and Dayhoff *et al*(1983) evaluated global alignment scores for randomized sequences (maintaining the amino acid composition and sequence length of the real sequences) using their log-odds scoring matrix at PAM250 and a constant gap penalty. The distribution of the resulting scores matched a normal distribution [8,9]. Webber and Barton used sequences belonging to different folds of the SCOP database whose percent identity to each other is less than 40 to analyze the distribution of global alignment z-scores between sequences [10]. They found that the gamma distribution describes the distribution of z-scores better than either the normal or Gumbel distribution.

The determination of homolog is also affected by the reference datasets used for statistical estimation. Lipman *et al *analyzed the distribution of scores among 100 vertebrate nucleic acid sequences and compared these scores with randomized sequences prepared in different ways [11]. When the randomized sequences were prepared by shuffling the sequence to conserve base composition, the standard deviation was approximately one-third less than the distribution of scores of the natural sequences, thus leading to an overestimate of the significance if such randomized sequences were used for a significant test. When sequences were locally shuffled for randomization, the standard deviation was similar to that of the natural sequences.

In this research, we chose 6 different ways to generate random and real but unrelated protein sequences as reference datasets for significance estimation. Four scoring matrices were applied for global alignments. The PAM120 and PAM250 matrices were selected because they imply different evolutionary time [8], and the BLOSUM50 and BLOSUM62 matrices were selected for their sound performance in database search [12,13]. Most alignments were carried out with the affine gap penalty (see Methods) as it penalizes less for additional gaps, which is more reasonable. The resulting alignment scores were then fitted with three distributions to obtain the statistical characteristic of the global alignment scores.

## Results

### Derivation of distributions with different sequence sets

We have generated random and real but unrelated sequences in six different ways as reference datasets. The datasets are abbreviated as ***LAC***, ***LCA***, ***GS***, ***LS***, ***SMP ***and ***RUS ***sequences respectively. The definition of the abbreviations can be found in the List of Abbreviations Used below.

In general the three-parameter gamma distribution performs the best in the goodness of fit test with the distribution of global alignment scores. When the shape parameter of the gamma distribution is sufficiently large, the gamma distribution closely approximates a normal distribution. Thus the normal distribution agrees with the data as well. The Gumbel distribution deviates from the alignment score distribution over the majority of the score frequencies, however its performance gets a little better for the ***LS ***sequence set than for the other sequence sets.

**Figure 1:**
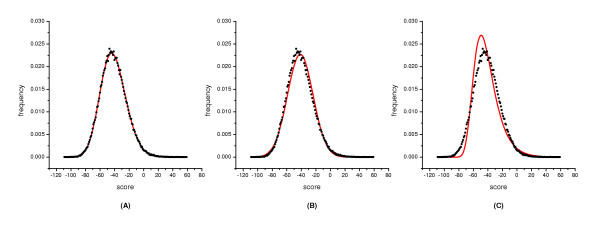
**Distribution of scores from global alignments of the *GS *sequence sets of 300aa long. **The alignments were carried out with the BLOSUM62 matrix and an affine gap penalty of -7 and -1. The distribution of the scores was fitted with three distribution curves indicated by the solid line. (A) The scores were fitted with the gamma distribution. The fitted parameters are: *γ *= 44.7296, *λ *= 0.381642, *μ *= 158.666, *χ*^2 ^value is 14.7643 with d.f. = 18; (B) The scores were fitted with the normal distribution. The fitted parameters are: *σ *= 17.5243, *μ *= -41.4629; d.f. = 19, *χ*^2 ^= 729.532; (C) The scores were fitted with the Gumbel distribution. The fitted parameters are: *β *= 13.66367, *μ *= -49.3489; d.f. = 19, *χ*^2 ^= 10563.8.

The distributions of global alignment scores of the ***LAC***, ***LCA ***and ***GS ***sequence sets are similar (Figure 1). The three-parameter gamma distribution defines the empirical distributions extremely well. The fitting quality of the normal distribution is better than that of the Gumbel distribution, but both of them diverged from the majority of the global alignment frequencies.

**Figure 2:**
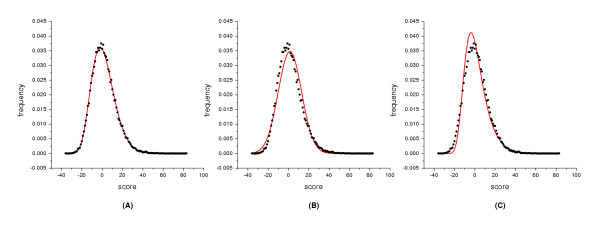
**Distribution of scores from global alignments of the *LS *sequence set whose sequence length is 200aa. **The alignments were carried out with the PAM250 matrix and an affine gap penalty of -10 and -2. The sequences were permutated within windows of 10 residues. The distribution of the scores was fitted with three distribution curves indicated by the solid line. (A) The scores were fitted with the gamma distribution. The fitted parameters are: *γ *= 11.5053, *λ *= 0.296252, *μ *= -37.4408, *χ*^2 ^value is 15.6515 with d.f. = 11; (B) The scores were fitted with the normal distribution. The fitted parameters are: *σ *= 11.4495, *μ *= 1.39533; d.f. = 12, *χ*^2 ^= 815.04; (C) The scores were fitted with the Gumbel distribution. The fitted parameters are: *β *= 8.927137, *μ *= -3.75695; d.f. = 12, *χ*^2 ^= 1001.89.

For the ***LS ***sequence sets the majority of the empirical score frequencies disagree with the normal or Gumbel distributions, whereas the three-parameter gamma distribution describes the alignment data extremely well (Figure 2). It also can be seen that the performance of the Gumbel distribution is better with this sequence set than with the others.

**Figure 3:**
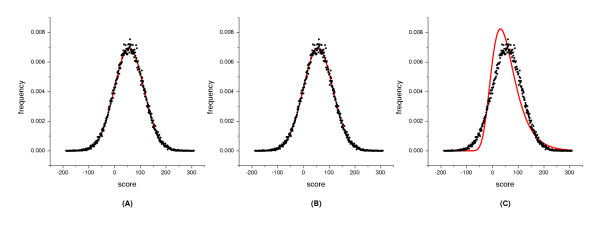
**Distribution of scores from global alignments of the SMP sequence set of 300aa long. **The alignments were carried out with the PAM120 matrix and an affine gap penalty of -16 and -4. The distribution of the scores was fitted with three distribution curves indicated by the solid line. (A) The scores were fitted with the gamma distribution. The fitted parameters are: *γ *= 3294940, *λ *= 31.7263, *μ *= -103798, *χ*^2 ^value is 10.8053 with d.f. = 12; (B) The scores were fitted with the normal distribution. The fitted parameters are: *σ *= 57.2142, *μ *= 56.5328; d.f. = 13, *χ*^2 ^= 11.0862; (C) The scores were fitted with the Gumbel distribution. The fitted parameters are: *β *= 44.6098, *μ *= 30.7864; d.f. = 13, *χ*^2 ^= 21244.1.

The distribution of alignment scores of the ***SMP ***sequence set is quite different. The empirical score distribution of sequences generated with the PAM120 mutation probability matrix and aligned with the PAM120 log-odds scoring matrix is different from those generated with the PAM250 matrix. Both the gamma and normal distributions fit the score frequencies of the former well (Figure 3), whereas for the latter, the normal distribution disagrees with the majority of the score curve.

**Figure 4:**
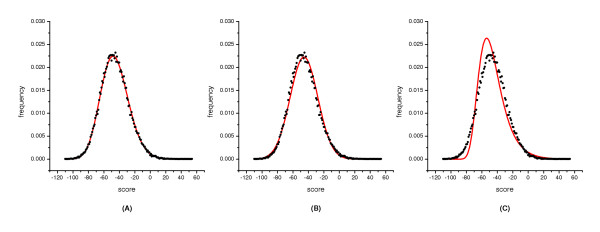
**Distribution of scores from global alignments of the RUS sequence set of 100aa long. **The alignments were carried out with the BLOSUM50 matrix and an affine gap penalty of -10 and -2. This sequence set include 300 sequences whose E-value to each other is greater than 10. The distribution of the scores was fitted with three distribution curves indicated by the solid line. (A) The scores were fitted with the gamma distribution. The fitted parameters are: *γ *= 53.0052, *λ *= 0.406777, *μ *= -176.022, *χ*^2 ^value is 13.7903 with d.f. = 10; (B) The scores were fitted with the normal distribution. The fitted parameters are: *σ *= 17.898, *μ *= -45.717; d.f. = 11, *χ*^2 ^= 308.925; (C) The scores were fitted with the Gumbel distribution. The fitted parameters are: *β *= 13.95498, *μ *= -53.771; d.f. = 11, *χ*^2 ^= 4659.8.

Most of the score distributions of the ***RUS ***sequence set agree well with the gamma distribution, no matter what the sequence length is. Although there are occasions when no distribution agrees perfectly well with the score distribution, the three-parameter gamma distribution is still the closest to the empirical distribution (Figure 4).

**Figure 5:**
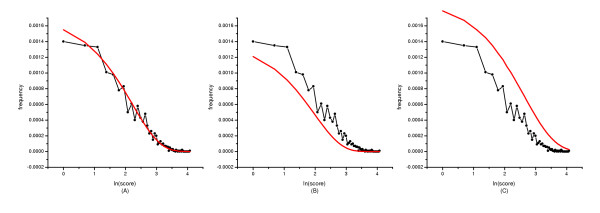
**Plots of the tail of the global alignment optimal score distribution. **The score frequencies were plotted against the natural logarithm of scores at the tail of the distribution of Figure 1. The three theoretical distributions were indicated in solid lines. The score distribution was fitted with (A) the three-parameter gamma distribution; (B) the normal distribution; and (C) the Gumbel distribution.

In database search, it is always the sequences, with higher scores (i.e., tail of the distribution), are of interest. So the score frequencies were also plotted against the natural logarithm of scores at the tail of the distribution (Figure 5). It shows clearly that the gamma distribution outperforms both the normal and Gumbel distributions even at the tails of those distributions.

### The effect of sequence length on the theoretical distribution

We chose the ***LAC ***sequence set to analyze the impact of the sequence length on the gamma distribution because the amino acid composition of the ***LAC ***sequence set is the same. The result shows that the shape parameter of the fitted gamma distribution increases and scale parameter decreases gradually as the sequence length increases, at the same time the performance of the normal distribution for curve fitting improves slowly (Table 2 and Table 3).

**Table 2:** Fitting of the global alignment scores aligned with affine gap penalty. All sequences were generated with the *LAC *approach with different sequence lengths and the alignments were carried out with the BLOSUM62 matrix and an affine gap penalty of -7/-1. Global alignment scores were fitted to the gamma and normal distribution respectively.

sequence	fitted gamma distribution						fitted normal distribution		score	
			
length	*γ*	*λ*	*μ*	d.f.	*χ*^2^	p-value	d.f.	*χ*^2^	mean	variance
50aa	41.00	0.63	-84.60	10	11.99	0.29	11	89.22	-19.19	104.36
100aa	49.16	0.55	-115.44	12	6.37	0.9	13	63.32	-25.40	164.90
200aa	52.24	0.44	-153.25	12	8.26	0.77	13	86.91	-33.58	274.14
300aa	58.60	0.41	-183.39	11	8.58	0.64	12	81.78	-39.55	353.04
400aa	56.17	0.36	-200.13	11	4.20	0.95	12	29.30	-45.36	426.44
500aa	66.17	0.36	-235.17	11	9.27	0.58	12	61.39	-50.31	516.48
600aa	68.51	0.34	-253.30	9	7.52	0.58	10	27.55	-54.30	578.04
700aa	73.11	0.34	-273.95	10	12.28	0.27	11	50.20	-58.67	633.89
800aa	74.73	0.32	-295.61	11	12.00	0.36	12	55.39	-62.46	727.41
1200aa	96.17	0.32	-376.88	12	7.52	0.82	13	26.49	-76.42	938.75

**Table 3:** Fitting of the global alignment scores aligned with constant gap penalty. All sequences were generated with the *LAC *approach with different sequence lengths and the alignments were carried out with the PAM250 matrix and a constant gap penalty of -10. Global alignment scores were fitted to the gamma and normal distribution respectively.

sequence	fitted gamma distribution						fitted normal distribution		score	
			
length	*γ*	*λ*	*μ*	d.f.	*χ*^2^	p-value	d.f.	*χ*^2^	mean	variance
50aa	156.20	0.90	-201.46	10	9.19	0.51	11	14.56	-25.97	197.16
100aa	113.08	0.58	-237.29	10	9.38	0.49	11	29.91	-41.55	338.82
200aa	341.64	0.78	-504.83	11	10.78	0.45	12	20.40	-68.55	557.15
300aa	110.45	0.39	-376.02	10	9.86	0.45	11	18.42	-91.93	730.71
400aa	110.74	0.35	-431.71	11	9.05	0.59	12	19.41	-114.46	908.81
500aa	158.16	0.38	-551.37	12	14.69	0.26	13	15.67	-136.15	1090.13
600aa	118.10	0.31	-537.57	11	11.67	0.39	12	30.80	-157.00	1226.38
700aa	118.40	0.29	-582.49	10	9.85	0.45	11	12.88	-178.59	1377.88
800aa	129.03	0.30	-635.51	11	7.47	0.74	12	33.63	-198.74	1478.53
1200aa	131.35	0.25	-798.05	9	11.07	0.26	10	12.83	-278.65	2053.78

### The difference of window size for local shuffling

The impact of the window size of local shuffling approach on the gamma distribution was also studied (Table 4). The result shows that when the window size increases, the shape parameter of the fitted gamma distribution decreases and the fitting performance of the normal distribution gets worse.

**Table 4:** Fitting of the global alignment scores of the *LS *sequence set generated with different window sizes. The sequence length is 200aa and all alignments were carried out with the BLOSUM62 matrix with an affine gap penalty of -7/-1. Global alignment scores were fitted to the gamma and normal distribution respectively.

window	fitted gamma distribution						fitted normal distribution		score	
			
size	*γ*	*λ*	*μ*	d.f.	*χ*^2^	p-value	d.f.	*χ*^2^	mean	variance
5aa	13.90	0.39	-40.14	9	9.82	0.36	10	239.33	-4.73	90.14
10aa	11.19	0.37	-29.01	10	12.13	0.28	11	236.91	0.99	80.40
15aa	10.60	0.35	-20.57	8	9.31	0.32	9	277.47	9.60	85.90
20aa	10.28	0.36	-16.85	13	17.04	0.19	14	334.87	11.93	80.56

### The impact of scoring scheme

The effects of the four scoring matrices are minor on the distribution of global alignment scores. The only exception is the empirical distribution of the ***SMP ***sequence set aligned with the PAM120 log-odds scoring matrix, in which the normal distribution performs as well as the gamma distribution.

## Discussion

Dayhoff *et al *(1978) and Dayhoff *et al *(1983) evaluated global alignment scores for randomized sequences generated as our ***LCA ***sequence set using the PAM250 log-odds scoring matrix and a constant gap penalty [8,9]. The distribution of the resulting scores matched a normal distribution. We tried both constant and affine gap penalty for the ***LCA ***sequence set and found that the distribution of optimal scores of the ***LCA ***sequence set agrees better with the gamma distribution than with the normal distribution.

Webber and Barton took sequences of different folds with less than 40 percent identity from the SCOP database for global alignments and fitted the z-scores to peak distributions [10]. They found that the gamma distribution describes the alignment scores between different folds better than either the normal or Gumbel distribution. The problem is that the derivation of z-scores requires additional alignments to be calculated, and the z-score carries with it an implicit and possible incorrect assignment of significance by the normal distribution.

The score distribution of the ***SMP ***sequence set simulated from the evolution of the ancestor sequence at PAM120 is an exception. The fitted shape parameter of the gamma distribution is very large, and the normal distribution fits with the data equally well. This is because sequences generated with the PAM120 mutation probability matrix are around 40 percent similar with each other, so they can hardly be viewed as random sequences for distributional statistical analysis.

This study specifies three-parameter gamma distribution as the theoretical distribution of global alignment scores of random protein sequences. It could be used for the inference of homology between sequences whose relationship is unknown through significance evaluation. One issue worth exploring further is to formulate a function taking sequence length, scoring scheme and amino acid composition into account so that rapid statistics conclusion is reached.

## Conclusion

The global alignment optimal scores have been regarded as normal [7] or Gumbel distributed [8]. We have shown here that the normal distribution holds only for sequences simulated with the PAM120 matrix, while the Gumbel distribution disagrees with the optimal alignment score frequencies for all sequence sets in this research. The study shows that the three-parameter gamma distribution describes the random sequence alignment scores better than the normal or Gumbel distribution. The normal distribution performs as well as three-parameter gamma distribution when the shape parameter of the gamma distribution is sufficiently large.

## Methods

### Dataset

The SCOP (Structural Classification of Proteins) database [14] provides a detailed and comprehensive description of the structural and evolutionary relationships between all proteins whose structure is known [15]. The classification is on hierarchical levels that embody the evolutionary and structural relationships. The hierarchy of the database from top to bottom is fold, superfamily and family. Proteins that share clear evolutionarily relationship are clustered in families, those that have low sequence identities but whose structural and functional features suggest that a common evolutionary origin is probable are placed together in superfamilies. Proteins are defined as having a common fold if they have the same major secondary structures in the same arrangement and with the same topological connections. The SCOP 1.65 (released on December 2003) was used in this study. It contains 40,452 domains organized in 2,327 families, 1,294 superfamilies and 800 folds. These domains correspond to 20,619 entries in the Protein Data Bank (PDB) [16].

The amino acid compositions of all the sequences in the SCOP 1.65 were calculated as shown in Table 1. The amino acid composition in Table 1 was taken as the average amino acid composition of proteins for random sequence generation.

**Table 1:** The amino acid composition of proteins in SCOP 1.65

amino acid	frequency	amino acid	frequency	amino acid	frequency	amino acid	frequency
Aln	0.0819	Gln	0.0372	Leu	0.0871	Ser	0.0607
Arg	0.0489	Glu	0.0634	Lys	0.0593	Thr	0.0582
Gly	0.0775	Met	0.0216	Asn	0.0444	Asp	0.0577
His	0.0233	Trp	0.0150	Tyr	0.0358	Cys	0.0151
Phe	0.0397	Pro	0.0466	Val	0.0709	Ile	0.0557

10 pairs of sequences with different sequence lengths- 50aa, 100aa, 200aa, 300aa, 400aa, 500aa, 600aa, 700aa, 800aa and 1200aa-were randomly chosen from the SCOP database to be managed with different approaches described below. The SCOP identifies of the 10 pairs of sequences are: d1aqt_1 a.2.10.1, d1gjja1 a.140.1.1, d1foka3 a.4.5.12, d1mk7d1 a.11.2.1, d1hx9a1 a.102.4.1, d1h6pb_ a.146.1.1, d1qgjb_ a.93.1.1, d1lj8a1 a.100.1.9, d1fppb_ a.102.4.3, d1jdpb_ c.93.1.1, d1eswa_ c.1.8.1, d1jv1a_ c.68.1.5, d1dhx_1 b.121.2.2, d1jqkf_ e.26.1.2, d2fcpa_ f.4.3.3, d1br2a2 c.37.1.9, d1qgra_ a.118.1.1, d1jqna_ c.1.12.3, d1i50b_ e.29.1.2, d1muka_ e.8.1.4.

### Sequence randomization approaches

#### 1) Maintaining the sequence length and average amino acid composition (LAC)

Sequences were generated as strings of independent characters where the amino acid in any position is proportional to its composition in proteins (Table 1) with a given sequence length.

#### 2) Maintaining the sequence length and the amino acid composition of the authentic sequences (LCA)

The amino acid compositions of the two authentic sequences were calculated and random sequences were generated retaining both the distributions of the amino acid composition and the sequence length of the original sequences.

#### 3) Global shuffling or permutation (GS)

Each residue in the authentic sequences is randomly repositioned anywhere in the sequence, so that both the amino acid composition and sequence length were maintained.

#### 4) Local shuffling or permutation (LS)

The sequence is broken into *n/w *windows (*n *denotes the length of the sequence and *w *is the length of the window, typically 5–20 residues) and the residues in each window are randomly shuffled. Both the sequence length and the local amino acid composition are retained with this approach. We selected four window sizes-5aa, 10aa, 15aa and 20aa- to compare their effects on the statistical distributions.

#### 5) Simulation of the mutational process (SMP)

To get sequences of a known evolutionary distance, we simulated the mutational process of the ancestor sequence. First, the PAM1 mutation probability matrix is multiplied by itself 120 or 250 times to get the PAM120 and PAM250 mutation probability matrices. The matrices provide information about each amino acid stayed unchanged or substituted by any other one at the given evolutionary distance. Then the fate of each residue in the new sequence was determined according to the PAM120 or PAM250 mutation probability matrix through computer simulation [8].

#### 6) Real but unrelated sequences (RUS)

The SCOP database also provides filtered sub datasets selected with different criteria, such as sequence identity percentage, E-value, or different hierarchy representatives [17]. We chose three subsets, one in which the sequences are less than 10 percent identity to each other, another with sequences whose E-value with one another is greater than 10, and the third with 800 sequences each represents one fold in the SCOP 1.65.

The sequence lengths in the three subsets vary from 23aa to 1504aa. As global alignment between sequences of highly different sequence lengths is not appropriate, we extracted sequences of similar length in each filtered sequence set further. 300 sequences of 50aa, 100aa, 200aa, 300aa and 400aa were extracted respectively from each of the filtered sequence set, and the tails of longer ones were cut off.

### Alignment algorithm

We wrote a C program for all the global alignments in this study. The program implements the Needleman-Wunsch dynamic programming algorithm [18] and penalized on end gaps.

For the ***LAC ***and ***SMP*** sequence sets, two sequences were generated at a time and global alignments were carried out between them, this process was repeated 10,000 times. For the ***LCA***, ***GS ***and ***LS ***sequence sets the first sequence was aligned with 5,000 randomizations of the second, then vice versa. Global alignments were carried out between every pair of the 300 sequences for the ***RUS ***sequence set.

### Scoring scheme

As the evolutionary distance of the ***SMP ***sequence set is known, the scoring matrix matching the giving evolutionary time was chosen for the alignments. For other sequence sets two matrices, the PAM120 and PAM250 from the PAM series and another two, the BLOSUM50 and BLOSUM62 from the BLOSUM series were employed. The respective gap open/extension penalty combination for the PAM120 is -16/-4, PAM250 -11/-1, BLOSUM50 -10/-2, BLOSUM62 -7/-1, as recommended by Vingron and Waterman, Mount, and Pearson [6,19,20]. Gap extension penalty is added for the first gap.

We also used constant gap penalty of -10 for the PAM250 matrix for the ***LAC ***and ***LCA ***sequence sets.

### Curve fitting

Optimal global alignment scores of the different sequence sets aligned with different scoring scheme were fitted with the Gumbel, normal and three-parameter gamma distributions respectively. Methods of moments were used for the estimation of the parameters of the optional distributions.

The probability density function of the gamma distribution is given as

f(x)=λγ(x−μ)γ−1e−λ(x−μ)Γ(γ)
 MathType@MTEF@5@5@+=feaafiart1ev1aaatCvAUfKttLearuWrP9MDH5MBPbIqV92AaeXatLxBI9gBaebbnrfifHhDYfgasaacH8akY=wiFfYdH8Gipec8Eeeu0xXdbba9frFj0=OqFfea0dXdd9vqai=hGuQ8kuc9pgc9s8qqaq=dirpe0xb9q8qiLsFr0=vr0=vr0dc8meaabaqaciGacaGaaeqabaqabeGadaaakeaacqWGMbGzcqGGOaakcqWG4baEcqGGPaqkcqGH9aqpdaWcaaqaaiabeU7aSnaaCaaaleqabaGaeq4SdCgaaOGaeiikaGIaemiEaGNaeyOeI0IaeqiVd0MaeiykaKYaaWbaaSqabeaacqaHZoWzcqGHsislcqaIXaqmaaGccqWGLbqzdaahaaWcbeqaaiabgkHiTiabeU7aSjabcIcaOiabdIha4jabgkHiTiabeY7aTjabcMcaPaaaaOqaaiabfo5ahjabcIcaOiabeo7aNjabcMcaPaaaaaa@4E19@

where *γ *(*γ *> 0) is the shape parameter, *λ *(*λ *> 0) is the scale parameter, and *μ *(*x - μ *≥ 0) is the location parameter.

The normal distribution is given as

f(x)=12πσe−(x−μ)22σ2
 MathType@MTEF@5@5@+=feaafiart1ev1aaatCvAUfKttLearuWrP9MDH5MBPbIqV92AaeXatLxBI9gBaebbnrfifHhDYfgasaacH8akY=wiFfYdH8Gipec8Eeeu0xXdbba9frFj0=OqFfea0dXdd9vqai=hGuQ8kuc9pgc9s8qqaq=dirpe0xb9q8qiLsFr0=vr0=vr0dc8meaabaqaciGacaGaaeqabaqabeGadaaakeaacqWGMbGzcqGGOaakcqWG4baEcqGGPaqkcqGH9aqpdaWcaaqaaiabigdaXaqaamaakaaabaGaeGOmaiJaeqiWdahaleqaaOGaeq4WdmhaaiabdwgaLnaaCaaaleqabaGaeyOeI0YaaSaaaeaacqGGOaakcqWG4baEcqGHsislcqaH8oqBcqGGPaqkdaahaaadbeqaaiabikdaYaaaaSqaaiabikdaYiabeo8aZnaaCaaameqabaGaeGOmaidaaaaaaaaaaa@4516@

where *μ *is the location parameter and *σ *(*σ *> 0) is the scale parameter.

The Gumbel distribution, given as

f(x)=1βexp⁡{−exp⁡[−(x−μ)β]−x−μβ}
 MathType@MTEF@5@5@+=feaafiart1ev1aaatCvAUfKttLearuWrP9MDH5MBPbIqV92AaeXatLxBI9gBaebbnrfifHhDYfgasaacH8akY=wiFfYdH8Gipec8Eeeu0xXdbba9frFj0=OqFfea0dXdd9vqai=hGuQ8kuc9pgc9s8qqaq=dirpe0xb9q8qiLsFr0=vr0=vr0dc8meaabaqaciGacaGaaeqabaqabeGadaaakeaacqWGMbGzcqGGOaakcqWG4baEcqGGPaqkcqGH9aqpdaWcaaqaaiabigdaXaqaaiabek7aIbaacyGGLbqzcqGG4baEcqGGWbaCcqGG7bWEcqGHsislcyGGLbqzcqGG4baEcqGGWbaCcqGGBbWwdaWcaaqaaiabgkHiTiabcIcaOiabdIha4jabgkHiTiabeY7aTjabcMcaPaqaaiabek7aIbaacqGGDbqxcqGHsisldaWcaaqaaiabdIha4jabgkHiTiabeY7aTbqaaiabek7aIbaacqGG9bqFaaa@52D0@

where *μ *is the location parameter and *β *(*β *> 0) is the scale parameter.

The *χ*^2 ^goodness of fit test was used to evaluate the fitting result. The degree of freedom for the fitting with gamma distribution is the number of intervals subtracts 4, while for normal and Gumbel distribution is the number of intervals subtracts 3.

## List of abbreviations used

***LAC: ***Maintaining the sequence length and average amino acid composition

***LCA: ***Maintaining the sequence length and the amino acid composition of the authentic sequences

***GS: ***Global shuffling or permutation

***LS: ***Local shuffling or permutation

***SMP: ***Simulation of the mutational process

***RUS: ***Real but unrelated sequences

## Authors' contributions

HP developed the code, tested program performance, analyzed the results, and drafted the manuscript. JT proposed many additional suggestions for improving algorithm performance. SC supported the research and improved the writing. ST conceived of the study and participated in its design and coordination. All authors read and approved the final manuscript.
